# Physicochemical Characterization, Rheological Properties, and Antimicrobial Activity of Sodium Alginate-Pink Pepper Essential Oil (PPEO) Nanoemulsions

**DOI:** 10.3390/foods13193090

**Published:** 2024-09-27

**Authors:** Mariah Almeida Lima, Juliana Carusi, Liliana de Oliveira Rocha, Renata Valeriano Tonon, Rosiane Lopes Cunha, Amauri Rosenthal

**Affiliations:** 1 Food Technology Department, Institute of Technology, University Federal Rural of Rio de Janeiro, Seropedica 23890-000, RJ, Brazil; 2 Department of Food Science and Nutrition, School of Food Engineering, University of Campinas (UNICAMP), Campinas 13083-862, SP, Brazil; jucarusi@unicamp.br (J.C.); lrocha@unicamp.br (L.d.O.R.); 3 Embrapa Food Technology, Rio de Janeiro 23020-470, RJ, Brazil; renata.tonon@embrapa.br (R.V.T.); amauri.rosenthal@embrapa.br (A.R.); 4 Department of Food Engineering and Technology, School of Food Engineering, University of Campinas (UNICAMP), Campinas 13083-862, SP, Brazil; rosiane@unicamp.br

**Keywords:** pink pepper essential oil, nanoemulsion, microfluidization, antimicrobial activity, sodium alginate

## Abstract

Essential oils (EOs) have antimicrobial properties, but their low solubility in water and strong flavor pose challenges for direct incorporation into food, as they can negatively impact organoleptic properties. To overcome these issues, strategies such as oil-in-water (O/W) nanoemulsions have been developed to improve EO dispersion and protection while enhancing antimicrobial efficacy. The objective of this study was to create sodium alginate-pink pepper essential oil (PPEO) nanoemulsions using microfluidization. Various formulations were assessed for physicochemical, physical, and antimicrobial properties to evaluate their potential in food applications. The microfluidized emulsions and nanoemulsions had droplet sizes ranging from 160 to 443 nm, polydispersity index (PdI) ranging from 0.273 to 0.638, and zeta potential (ζ) ranging from −45.2 to 66.3 mV. The nanoemulsions exhibited Newtonian behavior and remarkable stability after 20 days of storage. Antimicrobial testing revealed effectiveness against *Staphylococcus aureus* and *Listeria monocytogenes*, with minimum inhibitory concentrations (MIC) of 200 µg/mL for both microorganisms and minimum bactericidal concentrations (MBC) of 800 µg/mL and 400 µg/mL, respectively, proving that encapsulation of PPEO in nanoemulsions significantly increased its antibacterial activity. These results present the possibility of using PPEO nanoemulsions as a more effective natural alternative to synthetic preservatives in food systems.

## 1. Introduction

The demand for safe and high-quality foods is increasing among consumers. In this regard, the food sector has endeavored to offer safer and more natural alternatives as substitutes for synthetic and chemical antimicrobial agents, given the evidence indicating their adverse effects on human health [1,2,3]. Thus, natural preservatives derived from diverse plant components, including leaves, roots, fruits, and seeds, have been utilized to fulfill consumer demand for more natural foods [4]. Essential oils (EOs) are metabolites secondary to aromatic vegetal species that act naturally to protect against microbial and insect attacks [5]. They are acknowledged with the designation Generally Recognized as Safe (GRAS) for applications in foodstuffs. Their role as an ingredient is mainly as a substitute for synthetic preservatives due to their antioxidant and antimicrobial activities [6]. Pink pepper (*Schinus terebinthifolius* Raddi) is a plant indigenous to Brazil that is found in South and Central America, Europe, Asia, and Africa. Pink pepper essential oil (PPEO) has been suggested to have antitumor, antioxidant, and antimicrobial activities [7]. Prior research has established that the chemical constituents are primarily limonene, myrcene, α-pinene, and β-pinene, with a minor concentration of sesquiterpenes, including D-germacrene [8]. The Antimicrobial effects of PPEO are thought to be due to the presence of either monoterpene or sesquiterpene hydrocarbons, as well as their oxygenated derivatives, including limonene and α-pinene [9]. The effects of PPEO as an antioxidant and antimicrobial agent have been studied in foods such as cheese and meats [10,11,12]. However, its nanoencapsulated form has been little studied in terms of its antimicrobial activity against pathogenic microorganisms. Due to their volatility, flavor, and low solubility, the direct addition of these and other EOs becomes a challenge as they may negatively affect the organoleptic characteristics of foods [13].

To mitigate these issues, encapsulating EOs in nanoemulsions may provide a promising alternative, addressing the challenges associated with their direct application in foods and enhancing their effectiveness when used in active coatings [6]. Oil-in-water (O/W) nanoemulsions are defined as colloidal dispersions composed of nanometric oil droplets (d < 200 nm) dispersed in an aqueous phase [14,15]. Nanoemulsions are highly stable with regard to gravitational processes, including coalescence, creaming, flocculation, and sedimentation. Nevertheless, they are susceptible to Ostwald ripening [16,17]. The Ostwald ripening describes the phenomenon whereby larger droplets in a dispersed system, such as an emulsion, grow at the expense of smaller droplets. This occurs due to the difference in solubility between small and large droplets, resulting in a phase separation and a subsequent decrease in system stability [18]. The low water solubility of essential oils facilitates Ostwald ripening, which can occur immediately following emulsion formation. Consequently, the combination of water-insoluble oils, such as sunflower oil, and emulsifiers, like lecithin, with essential oils can reduce surface tension, which impedes droplet growth due to the entropy of mixing effect that opposes Ostwald ripening [19]. The nanometric size of the oil droplets in nanoemulsions results in an increase in surface area, thereby enhancing the efficacy of the added compounds. This, in turn, permits a reduction in concentration (10–50%) while minimizing the potential for sensory alterations [13,20].

The combination of emulsifiers, surfactants, and stabilizers can facilitate the formation of the emulsion, in addition to improving its properties and increasing its stability [21]. Medium or long-chain triglycerides, such as corn and sunflower oil, are defined as “ripening inhibitors”, as they form a kinetic barrier, making EOs less soluble in water, reducing the rate at which smaller particles dissolve and reprecipitate into larger particles [22,23]. Soy lecithin is a natural emulsifier derived from the cell membrane of soybeans containing hydrophobic groups and alkyl side chains that make it amphiphilic, reducing the interfacial tension between the phases of the emulsion and improving its stability [24]. Polysorbates, also designated as Tweens, are non-ionic surfactants derived from sorbitan and are commonly utilized in the encapsulation of essential oils. Tweens 20 and 80, though derived from the same compound, possess distinct structures [25]. Tween 20 contains a side chain comprising lauric acid, whereas Tween 80 has oleic acid in its side chain, rendering it less hydrophilic than Tween 20 [26,27]. The structural differences between polysorbates allow them to stabilize nanoemulsions in different ways, which directly impacts the size and stability of the droplets.

Polysaccharide-based nanoemulsions, such as sodium alginate, incorporating EO as an antimicrobial agent, can be utilized to form edible active coatings. Using these coatings can increase shelf life and help prevent spoilage of food products. A number of studies report on various properties associated with polysaccharide-based nanoemulsions developed using microfluidization, as well as their applications in active films and coatings [28,29,30,31]. Khanzadi et al. [31] observed inhibition of microbial growth in trout fillets coated with sodium alginate nanoemulsion containing *Zataria multiflora* Boiss essential oil. Kazemeini, Azizian, and Adib [32] demonstrated that nanoemulsion comprising sodium alginate-*Trachyspermum ammi* essential oil, when used as a coating, was capable of inhibiting the growth of *Listeria monocytogenes* in turkey fillets even after 12 days of storage at 4 °C.

Despite a few studies focused on the nanoencapsulation of pink pepper essential oil (PPEO) in nanoemulsions, no studies have specifically evaluated the antimicrobial potential of these nanoemulsions against foodborne pathogenic microorganisms, which can be of great interest to the food industry. Additionally, the combination of sunflower oil and lecithin with surfactants may prove an effective strategy for obtaining stable nanoemulsions, preventing destabilization phenomena such as Ostwald ripening and creaming. The aim of this study was to develop sodium alginate-pink pepper essential oil (PPEO) nanoemulsions) using sunflower oil and lecithin as stabilizers and to characterize their physicochemical and rheological properties after microfluidization. Furthermore, the antimicrobial potential of these nanoemulsions was assessed against foodborne pathogenic microorganisms, specifically *Salmonella* sp., *Escherichia coli*, *Listeria monocytogenes*, and *Staphylococcus aureus*, aiming their application as active coatings in animal-based products, such as cheeses.

## 2. Materials and Methods

### 2.1. Material

PPEO was acquired from Ferquima (São Paulo, Brazil). The company provided the composition of the essential oil: γ-terpinene (32%), α-phellandrene (20%), δ-limonene (15%), α-pinene (10%), and ρ-cimene (4%). Surfactants Tween 20 and Tween 80 were purchased from Dinamica (São Paulo, Brazil). Sodium alginate was obtained from GastronomyLab^®^ (Brasília, Brazil). Mueller-Hinton Agar and Mueller-Hinton broth were obtained from OXOID^®^ (Basingstoke, UK). Soy lecithin and sunflower oil were kindly donated by Bremil (Arroio do Meio, RS, Brazil) and Cargill Global (Mairinque, SP, Brazil), respectively. Nanoemulsions were produced using ultrapure water from a Milli-Q Plus system. The bacteria employed in the investigation of antimicrobial activity study included *Listeria monocytogenes* ATCC 7644, *Staphylococcus aureus* ATCC 6538, *Escherichia coli* ATCC 11229, and *Salmonella* sp. (LMA 32—Unicamp internal code).

### 2.2. Nanoemulsion Preparation

The oil-in-water nanoemulsions were developed by keeping the concentration for all components fixed in relation to the amount of water. Based on the study by Salvia-Trujillo et al. [33], the amount of PPEO, sodium alginate, and non-ionic surfactant (Tween 20 or Tween 80) remained constant at 1.0% (*w*/*w*). In some formulations, sunflower oil (0.5 or 0.75% *w*/*w*) or soy lecithin (0.2% *w*/*w*) was also added. These concentrations were chosen from our preliminary experiments to be published elsewhere, based on the work of Ghaderi et al. [34] and Asadinezhad et al. [16] with modifications, where the concentrations of sunflower oil varied from 0.25 to 1% (*w*/*w*) and lecithin from 0.1 to 0.2% (*w*/*w*), and the concentrations selected were based on the smallest droplet size and the best physical stability of the nanoemulsions. The identification and composition of the formulations are presented in Table 1. For identification, the samples were prefixed with CE—(coarse emulsion), and the nanoemulsions were prefixed with M—(microfluidized) before the sample name.

The aqueous phase was produced by solubilizing sodium alginate and an emulsifier in ultrapure water heated to 70 °C under magnetic stirring until complete dissolution, followed by cooling at refrigeration temperature. Non-ionic surfactant was added to the aqueous phase. The emulsion was obtained by combining the aqueous and oil phases (PPEO, PPEO + sunflower oil or lecithin) in an Ultra Turrax^®^ T18 digital (S 18 N-19 G dispersing tool element with a diameter of 19 mm, IKA, Staufen, Germany) for a period of 5 min at 13,000 rpm at 20 °C. Nanoemulsion was produced by subjecting the coarse emulsion to a microfluidizer (LM20-20, Z-Type Interaction Chamber model H210Z, Microfluidics, Westwood, MA, USA) at 15,000 psi for a total of five cycles. Subsequently, the product was cooled in an ice-water bath at the outlet of the microfluidizer interaction chamber, ensuring that the sample temperature remained below 30 °C. Besides, the pH of all the samples was measured after microfluidization using a pH-meter (Metrohm 827 pH Lab, Metrohm, São Paulo, Brazil) with values of approximately 6.8 ± 0.2. Experiments were conducted in triplicate.

### 2.3. Physicochemical Properties of the Nanoemulsions

#### 2.3.1. Particle Size, Size Distribution, and Polydispersity Index

Particle size distribution of the emulsions and nanoemulsions was determined using a Zetasizer Nano-ZS (Malvern Instruments Ltd., Worcestershire, UK) at a wavelength of 633 nm and temperature 25 °C, with a backscatter detector of 173°. Prior to analysis, each sample was diluted with ultrapure water at a ratio of 1:99 in order to minimize multiple scattering effects. Size distribution was measured by setting the Mie theory, and a refractive index value of 1.46 was used for the disperse phase (pink pepper essential oil) and 1.33 for the continuous phase (water) [35]. Mean size and polydispersity index were evaluated based on the intensity particle size distribution. D10, D50, and D90 were derived from the volume-weighted distribution, indicating the size below which 10%, 50%, or 90% of all particles are located, respectively.

#### 2.3.2. ζ-Potential

The zeta (ζ) potentials (mV) were measured by electrophoretic light scattering using a Zetasizer Nano-ZS instrument (Malvern Instruments Ltd., Worcestershire, UK). Prior to analysis, the emulsions were diluted in ultrapure water using a dilution factor 1:9 (sample to solvent). Measurements were made on the pH of the samples (6.8 ± 0.2).

#### 2.3.3. Whiteness Index

The chromatic properties of emulsions and nanoemulsions were determined with a Hunter Lab colorimeter model UltraScanVIS (HunterLab, Reston, VA, USA) employing the CIE Lab scale (L*, a*, and b*) at room temperature. The device was calibrated with a standard white plate using the illuminant D65 and 10° observer angle settings, and the whiteness index (WI) value using the following Equation (1) [36]:

(1) $$WI=100-{\left((100-L^{*}\right)}^{2}+{{(a}^{*2}+b^{*2}))}^{0.5}$$

where L* determines the brightness of the color, a* determines the position between green and red, and b* determines the position between blue and yellow.

#### 2.3.4. Rheology Characterization

Emulsions and nanoemulsions flow curves were obtained using an AR1500ex rheometer (TA Instruments, Crawley, UK) equipped with double concentric cylinder geometry comprising an internal cylinder (external radius of 17.53 mm, internal radius of 16.02 mm) and an external cup (external radius of 18.45 mm, inner radius of 15.10 mm). A gap of 500 µm and the shear was adjusted within a range of 0 and 300 s^−1^. In order to eliminate any potential effects of shear time, the flow curves were generated using up-down-up cycles. Emulsions and nanoemulsions were evaluated just after their preparation. Triplicate measurements were taken at 25 °C. The data obtained from the third flow curve were modeled using the Newtonian and power-law (Equation (2)) fluid models:

(2) $$\sigma =k\cdot {\left(\dot{\gamma }\right)}^{n}$$

In this context, *σ* represents shear stress (Pa), *k* denotes the consistency index (Pa·s^n^), $\dot{\gamma }$ signifies the shear rate (s^−1^) and *n* is the flow behavior (dimensionless) [37].

#### 2.3.5. Emulsion Stability

The stability of the emulsions and nanoemulsions was evaluated in accordance with the methodology proposed by Ushikubo and Cunha [38], with modifications. Twenty-five milliliters of the emulsions were transferred to cylindrical glass tubes, which were then sealed and stored for a period of 30 days at a temperature of 25 °C. The percentage separation of the oil phase was expressed as a separation index (SI), calculated with Equation (3).

(3) $$SI\left(\%\right)=\left(\frac{H}{H_{0}}\right)\times 100$$

where H is the height of the separated oil phase, and H_0_ is the total height of the sample.

The stability of the emulsion was also measured by the average droplet diameter. Measurements were performed every 10 days for 4 weeks at a temperature of 25 °C.

#### 2.3.6. Optical Microscopy

The microstructure of emulsions and nanoemulsions was observed in an optical microscope (Axio Scope.A1, Carl Zeiss, Jena, Germany) with a 100× oil immersion objective lens. The images were captured using the AxioVision Rel. 4.8 software (Carl Zeiss, Germany). The microstructure of emulsion and nanoemulsions was observed after preparation.

#### 2.3.7. Oil Retention

The total oil retained in emulsions and nanoemulsions was quantified via hydrodistillation in a Clevenger apparatus in triplicate, in accordance with the methodology delineated by Garcia, Tonon, and Hubinger [39], with modifications. Firstly, 100 mL of the sample was transferred to a 500 mL round-bottomed flask. The distillation process was conducted for a period of 60 min, and the distilled oil volume was directly read directly into the Clevenger apparatus. The percentage of oil retention was calculated according to Equation (4):

(4) $$Oil\;retention\left(\%\right)=\left(\frac{V\times \rho }{m}\right)\times 100$$

The volume of oil, denoted V, is that collected following distillation process. The term ρ represents the density of pink pepper essential oil or the mixture (PPEO and sunflower oil), while m denotes the mass of essential oil added to the sample.

### 2.4. Antimicrobial Activity

#### 2.4.1. Agar Disk Diffusion

Firstly, the antimicrobial activity of non-encapsulated PPEO (control) and nanoemulsions was determined via the disk diffusion technique, as previously described by CLSI [40]. Additionally, solutions of Tween 20, Tween 80, and sodium alginate at a concentration of 1% (*w*/*w*) were also tested. The bacterial cultures were suspended in peptone water (0.1% *w*/*v*) to achieve a concentration of 10^8^ CFU/mL, which is equivalent to a 0.5 Mc Farland standard. A volume of 100 µL of the inoculum was distributed with a sterilized swab onto the surface of Petri dishes with Mueller-Hinton Agar (MH-Oxoid^®^) as the growth medium. After that, the sterilized paper disks positioned on the plate received 10 µL of sample solution (non-encapsulated PPEO or microfluidized emulsion). The Petri dishes underwent incubation at 37 °C (*E. coli*, *Salmonella* sp., and *S. aureus*) and 30 °C (*L. monocytogenes*) for 24 h. After the incubation period, the presence of the inhibition zone was confirmed and quantified using a digital caliper.

#### 2.4.2. Minimum Inhibitory and Bactericidal Concentrations

The broth technique was utilized to establish the minimal inhibitory concentration (MIC) and minimal bactericidal concentration (MBC), according to CLSI methodology [41]. The non-encapsulated PPEO (control) and nanoemulsions were diluted in Mueller-Hinton broth (MH-Oxoid^®^) supplemented with a Tween 80 solution (0.5% *v*/*v*) up to a concentration of 6400 µg/mL. Then, 5 mL of this solution was transferred to a glass tube containing 5 mL of pure Mueller-Hinton broth until 3200 to 25 µg/mL concentrations were obtained. A total of 250 µL of inoculum (10^8^ CFU/mL) was added to each tube, which was then incubated at 37 °C (*E. coli*, *Salmonella* sp., and *S. aureus*) and 30 °C (*L. monocytogenes*) for 24 h. Subsequently, the tubes were evaluated, and the MIC was defined as the highest dilution without cell growth (compared to the negative control) through visual analysis.

MBC was obtained through inoculation of 10 µL from each tube that exhibited no growth in the MIC test onto Tryptic Soy Agar (TSA) plates. The lowest concentration that did not demonstrate growth was identified as the minimum bactericidal concentration (MBC) following a 24-h incubation period at 37 °C and 30 °C, respectively.

### 2.5. Statistical Analysis

The data were subjected to statistical analyses using the R i386 software (version 3.6.1; R Foundation for Statistical Computing, Vienna, Austria) to verify the behavior of the samples through analysis of variance (ANOVA). When a statistical difference was observed (*p* < 0.05), a Tukey test was employed for further investigation into the disparity between the corresponding means.

## 3. Results and Discussion

### 3.1. Particle Size Distribution, PdI and ζ-Potential

Microfluidization broke the primary emulsion droplets (micrometer size) into smaller sizes in microfluidized emulsions (mean size lower than 400 nm). This can be clearly observed in the z-average and D50 values (Table 2). There is still a lack of consensus on the size that defines what is classified as a nanoemulsion and an emulsion. A droplet diameter of less than 200 nm is indicative of nanoemulsion [15,18,42]. Considering this classification, only samples M-T20, M-T80, and M-0.5T20 cannot be classified as nanoemulsions, considering the z-average and D50 values. Mechanical stress caused by microfluidization promotes the formation of smaller droplets that are promptly enveloped by surfactant molecules, thereby reducing interfacial tension [43]. A lower mean size indicates a higher stability of nanoemulsions in comparison to coarse emulsions. Additionally, the size significantly affects the physicochemical, rheological, antimicrobial, and sensory properties and stability of nanoemulsion formulations [44].

**Figure 1:**
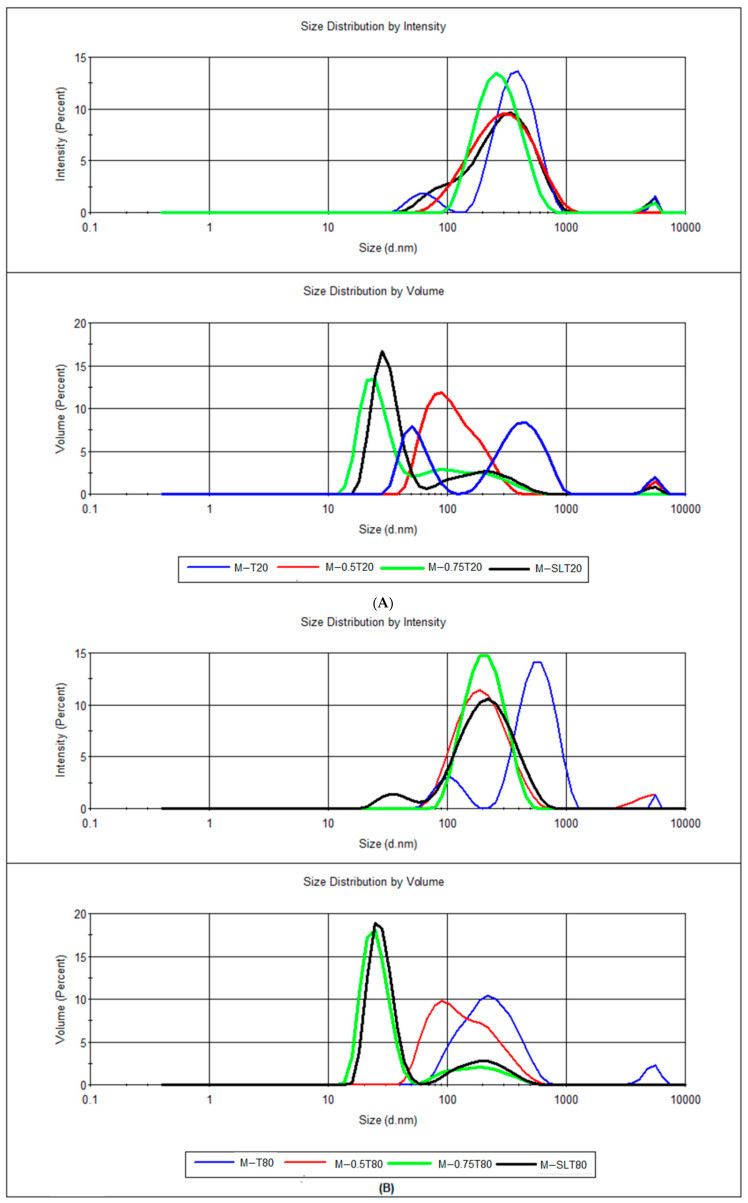
Particle size distribution (nm) of the sodium alginate-PPEO nanoemulsion expressed in intensity and volume stabilized by (**A**) Tween 20 and (**B**) Tween 80. M: microfluidized; T80: Tween 80; T20: Tween 20; 0.5: 0.5: 0.5% (*w*/*w*) sunflower oil; 0.75: 0.75% (*w*/*w*) sunflower oil; SL: soy lecithin.

The determination of particle size by DLS is predicated on the variation in the intensity of the laser beams that are scattered when they encounter the moving particles in a suspension [45]. This information is employed to ascertain the diffusion coefficient, which is used to calculate the hydrodynamic size in accordance with the Stoke-Einstein equation. DLS values are generated in relation to intensity; however, based on the intensity-weighted distribution, it is possible to generate volume-weighted and number-weighted distributions. According to the Rayleigh approximation, the intensity of the scattered light is proportional to the sixth power of the diameter (d^6^), being dominated by large particles that contribute disproportionately to the measured intensity, even when in smaller quantities. In contrast, the volume distribution is proportional to the third power of the diameter (d^3^), providing a more accurate representation of the material distribution and facilitating visualization of the smaller particles [46,47]. The particle size distribution showed a multimodal distribution in all nanoemulsions (Figure 1), which indicates a polydisperse distribution of particle sizes. The particle size distribution of the coarse emulsions can be found in the Supplementary Material (Figure S1).

The small peak observed around 5000 nm could be aggregates of biopolymer molecules and/or surfactants, as demonstrated by Artiga-Artigas et al. [48]. When formulating a nanoemulsion with a low concentration of essential oil, the surfactant adsorbed at the droplet interface can lead to interaction between the biopolymer chains. Alternatively, these chains may exhibit a preference for binding to unbound surfactant molecules in preference to those situated on the oil droplet surface [49]. Sodium alginate interacts with the surfactant chains surrounding the oil droplets, resulting in the induction of steric and/or electrostatic repulsion between the droplet interfaces. This interaction effectively prevents destabilization phenomena, including coalescence and gravitational separation [48]. Silva et al. [50] investigated the interfacial tension between the components of an emulsion comprising sacha inchi oil, sodium alginate, and polysorbates (Tween 20 and 80). The times required to reach equilibrium tension were approximately 600 s for both Tween 20 and 80 (1.0 wt%), indicating that the equilibrium tension was achieved due to the steric stabilization capacity of sodium alginate in conjunction with the rapid diffusion of polysorbate molecules. Moreover, in a system comprising sodium alginate and Tween, the surfactant exerts a dominant influence on the surface of the drop, given its superior active surface area in comparison to sodium alginate.

According to Salvia-Trujillo et al. [33], z-average in non-monodispersed distributions can lead to misinterpretation, and for this, D10, D50, and D90 values are also displayed. These values allowed us to observe the effect of the addition of another surfactant (lecithin) and sunflower oil. Sunflower oil acted as an Ostwald ripening inhibitor and soy lecithin as a co-surfactant, resulting in the generation of nanoemulsions with diminished droplet dimensions, which is in agreement with other studies [51,52]. Due to the increased surface area resulting from reduced droplet size and the greater water solubility of EO, nanoemulsions become more susceptible to Ostwald ripening, which occurs when smaller droplets merge from large ones, driven by the molecular propagation of the dispersed phase through the continuous phase [16,53]. Medium or long-chain triglycerides act by reducing the water solubility of EO, stabilizing the nanoemulsion by minimizing the propensity for larger droplets to form from smaller droplets, which is crucial to maintaining the desired properties and effectiveness of the formulation [23,54]. Low molecular mass surfactants, including polysorbate (Tweens) and lecithin, are promptly adsorbed at the oil-water interface during emulsification, leading to a particle size reduction [33]. Lecithin played a crucial role as a co-surfactant in nanoemulsion formulation, contributing to the reduction in droplet size. Its complex composition, primarily consisting of phospholipids such as phosphatidylinositol (PI), phosphatidylethanolamine (PE), and phosphatidylcholine (PC), along with other lipids, contributes to its effectiveness. The lamellar structures formed by these phospholipids, including mono- and bilayers, create a layer of protection around the dispersed droplets in nanoemulsion, preventing coalescence or fusion of the droplets, resulting in a decrease in droplet size and enhanced nanoemulsion stability [52,55].

Polydispersity index values are presented in Table 2. PdI is a parameter that denotes the range of the particle size distribution. Values below 0.3 indicate a narrow size distribution, suggesting enhanced droplet uniformity since less variation in emulsion droplet size may indicate better stability against the phenomena of coalescence or Ostwald ripening [56]. Regarding PdI, the only samples exhibiting indices below 0.3 were those containing 0.75% *w*/*w* sunflower oil (M-0.75T20 and M-0.75T80), which demonstrated superior kinetic stability outcomes over a 30-day storage period. The observed reduction in polydispersity index values may be attributed to the incorporation of sunflower oil, as the increase in the oil phase may facilitate a more uniform distribution of the surfactant (Tween) within the emulsion. Furthermore, sunflower oil comprises natural emulsifiers, including lecithin, which, due to its amphiphilic nature, provides stability to nanoemulsions by forming a stable layer at the oil-water interface. This contributes to a reduction in interfacial tension and the promotion of the development of smaller, more uniform droplets [57]. Moreover, the presence of phosphate groups facilitates the establishment of repulsive electrostatic interactions, which are essential for preventing coalescence [58]. The remaining samples exhibited higher PdI values, exceeding 0.3, indicative of a significant variability in the size of the emulsion droplets. This often renders them less stable, as the extensive range of droplet size renders them susceptible to destabilization phenomena, resulting in visual or creaming phase separation. As observed by Artiga-Artigas et al. [48], high PdI values may result from the re-coalescence phenomena, which can occur after the microfluidization process.

**Figure 2:**
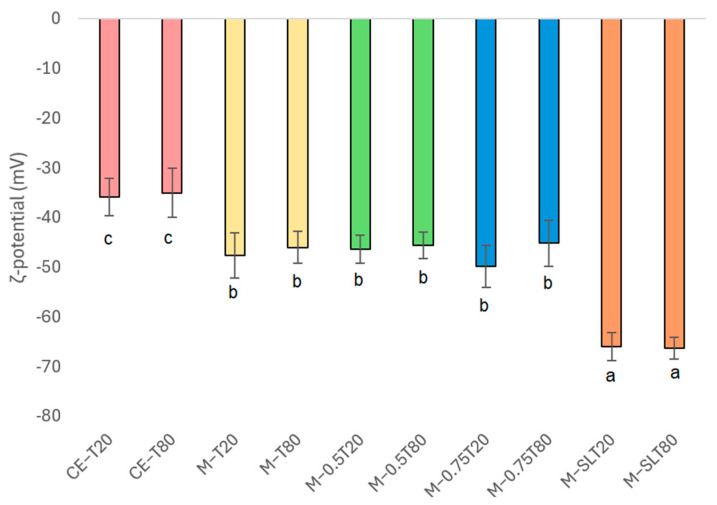
ζ-potential (mV) of coarse emulsions and nanoemulsions Significant differences within each column are indicated by different letters (*p* < 0.05) by the Tukey test. CE: coarse emulsion; M: microfluidized; T80: Tween 80; T20: Tween 20; 0.5: 0.5% (*w*/*w*) sunflower oil; 0.75: 0.75% (*w*/*w*) sunflower oil; SL: soy lecithin. The data are expressed as means values ± standard deviation.

Figure 2 presents ζ-potential values for sodium alginate-PPEO emulsions. All nanoemulsions displayed negative ζ-potentials, with a range of −35.0 mV to −66.3 mV. When the ζ-potential is less than −30 mV, the considerable electrical charge on the particles indicates that repulsive forces are the dominant force, contributing to the system stability. The negative ζ-potential values observed in every tested sample are attributed to the preferential adsorption of non-ionic surfactants, such as Tweens, onto the surface of the oil in conjunction with polymeric stabilizers, as is the case with sodium alginate [59]. The incorporation of sodium alginate into nanoemulsion formulations is instrumental in the electrostatic stabilization of these systems. This is due to the carboxylate and hydroxyl groups, which can readily dissociate in the hydrophilic phase and be adsorbed onto the oil droplet surface, thereby conferring sufficient negative charge to prevent destabilization phenomena [60]. Additionally, deprotonated alcohols (R-O-) and carboxylic acids (R′CO^2^-) are responsible for an increase in electron density that consequently results in higher negative ζ-potential values [28]. Tweens are vulnerable to oxidative breakdown brought on by exposure to light, oxygen, and heat, resulting in the formation of hydroperoxides, aldehydes, and carboxylic acids. This process introduces new functional groups, such as carboxyl groups, which ionize in the water phase, altering the surface of nanoemulsion droplets and increasing negative charge. Furthermore, the presence of impurities and ionic contaminants can also contribute to negative zeta potential values [61,62,63].

The nanoemulsion formulated with lecithin exhibited the lowest ζ-potential values, with M-SLT20 and M-SLT80 displaying −65.9 and −66.3 mV, respectively. These values are the result of lecithin phosphate groups and their negative charges that contribute to reducing the charge on the surface of the droplets, thereby increasing the negative ζ-potential [58].

### 3.2. Whiteness Index

**Figure 3:**
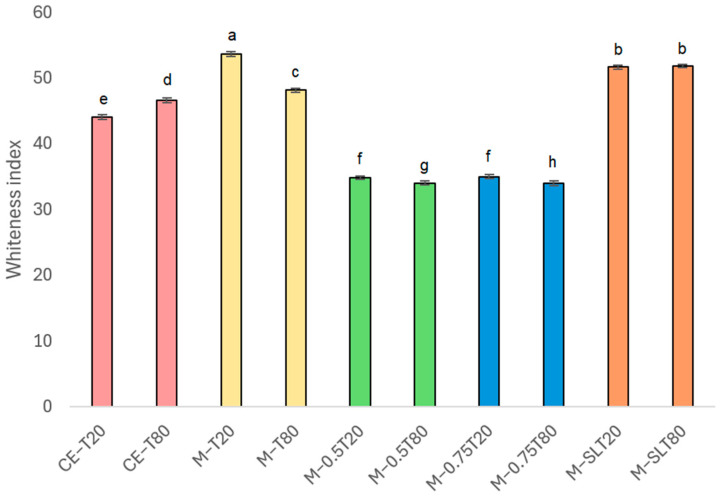
Whiteness index of coarse emulsions and nanoemulsions. Significant differences within each column are indicated by different letters (*p* < 0.05) by the Tukey test. CE: coarse emulsion; M: microfluidized; T80: Tween 80; T20: Tween 20; 0.5: 0.5% (*w*/*w*) sunflower oil; 0.75: 0.75% (*w*/*w*) sunflower oil; SL: soy lecithin. The data are expressed as means values ± standard deviation.

**Figure 4:**
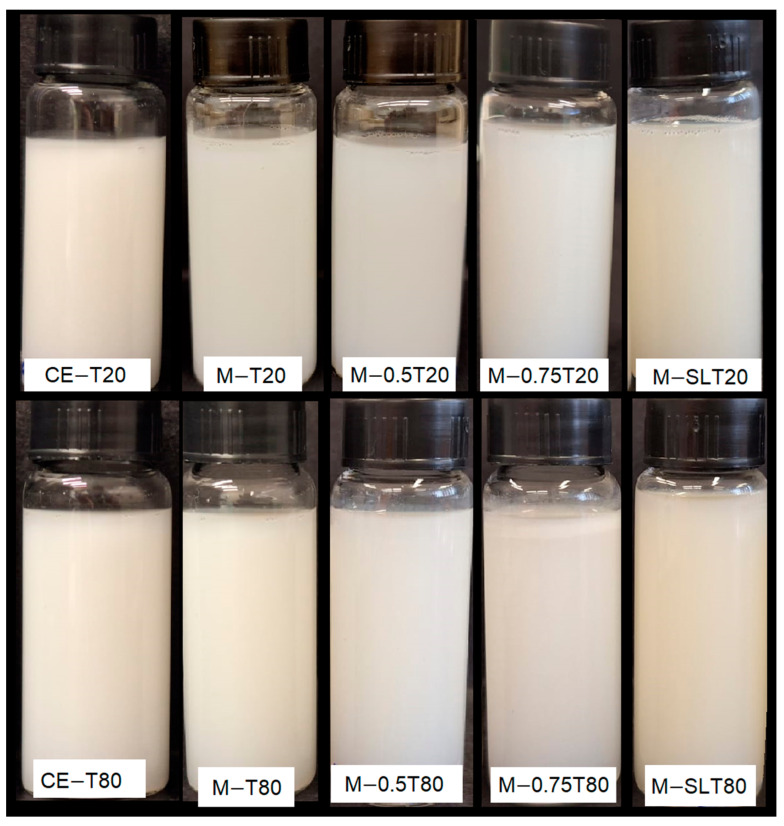
Images of sodium alginate-pink pepper essential oil (PPEO) emulsions and nanoemulsions. CE: coarse emulsion; M: microfluidized; T80: Tween 80; T20: Tween 20; 0.5: 0.5% (*w*/*w*) sunflower oil; 0.75: 0.75% (*w*/*w*) sunflower oil; SL: soy lecithin.

The Whiteness index (WI) is an important parameter employed to evaluate the optical properties of emulsions, reflecting the degree of light scattering by droplets within these systems, directly impacting their appearance and stability. In general, nanoemulsions are described as slightly cloudy systems, attributed to the weak scattering of light by their small droplets. As the size of the droplets increases, light scattering becomes more intense, making the emulsions appear more opaque [64]. Figure 3 presents the WI values of coarse and microfluidized emulsions. Despite the reduction in droplet size, all microfluidized emulsions and nanoemulsions presented an opaque visual appearance after microfluidization, and the whiteness index of some nanoemulsions was even higher than that of primary emulsions (Figure 4). Regardless of the surfactant, emulsions showed a similar behavior. Nanoemulsions with a lower oil content, M-T20, M-T80, M-SLT20, and M-SLT80, showed the highest WI values, which can be attributed to some factors associated with the composition. Firstly, the high polydispersity in the particle distribution shows a population size on the micrometer scale that may be aggregated alginate particles. Larger particles exhibit more intense light scattering compared to smaller ones, leading to amplified luminosity, opacity, and an enhanced whiteness index measurement for the nanoemulsions [14,33]. Additionally, the inclusion of soy lecithin, which exhibits a pronounced color, alters the overall hue of the nanoemulsion [65] and contributes to an increment in the WI value.

Notably, the addition of sunflower oil, M-0.5T20, M-0.5T80, M-0.75T20, and M-0.75T80, reduced WI values. Smaller average droplet size, lower PdI, and narrower size distribution are some of several factors that may influence the reduction of WI values. In nanoemulsions, smaller droplets have a reduced capacity to scatter light effectively compared to larger droplets, resulting in decreased whiteness [64]. Therefore, the combination of these factors leads to a weakened light scattering effect, reducing the perception of whiteness in samples [66]. Light scattering is most effective when the droplet size is closer to 380–780 nm (the wavelength of visible light). However, nanoemulsions typically comprise droplet sizes well below 200 nm, which results in reduced light scattering and a corresponding decrease in the whiteness index [67]. A lower polydispersity index indicates a more uniform droplet size distribution within the nanoemulsion, which, in turn, reduces the variability in light scattering. Consequently, there is a decrease in diffuse reflection and an associated reduction in the whiteness index. These characteristics contribute to a smoother, more consistent appearance, which is why nanoemulsions are used in products such as creams, gels, or liquid foods. In fact, the specific type and composition of the oil utilized and nanoemulsions formulation have a notable impact on the whiteness index of the emulsions. In their study, Salvia-Trujillo et al. [33] produced nanoemulsions using ten distinct essential oils, and only three nanoemulsions achieved transparency after microfluidization (150 MPa/3 cycles). This finding highlights the significant impact of formulation composition on the optical and visual characteristics exhibited by nanoemulsions.

### 3.3. Rheological Properties

**Figure 5:**
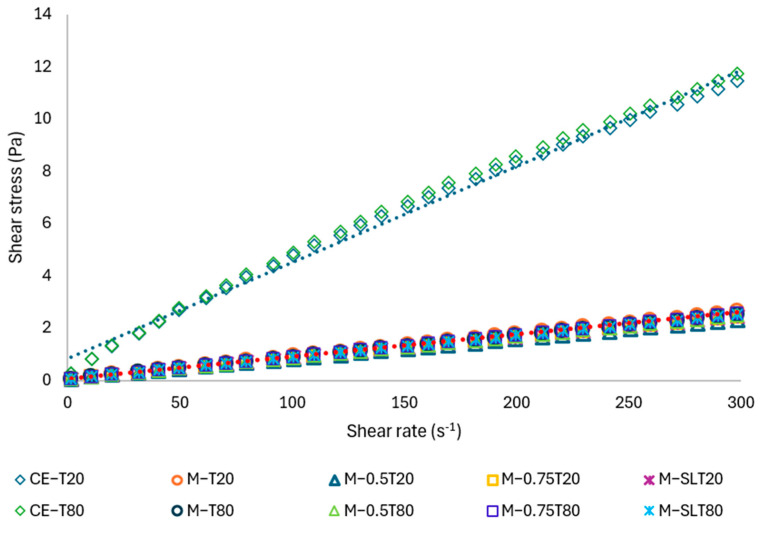
Flow curves of coarse emulsions and sodium alginate-PPEO nanoemulsions as a function of the shear rate. CE: coarse emulsion; M: microfluidized; Tween 80; T20: Tween 20; 0.5: 0.5% (*w*/*w*) sunflower oil; 0.75: 0.75% (*w*/*w*) sunflower oil; SL: soy lecithin.

The viscosity and concentration of the dispersed phase play a crucial role in the formation of nanometric emulsion droplets [68]. The sodium alginate-PPEO emulsions flow curves, shown in Figure 5, were well-fitted to the power-law model (Table 3). The coarse emulsions exhibited a flow index (n) of approximately 0.72 and evidenced pseudoplastic fluids behavior. The value of *n* is less than 1, which indicates pseudoplasticity or shear-thinning behavior, which is defined by a decrease in viscosity as the shear rate increases [69]. After the microfluidization process, all samples, regardless of the formulation, showed low viscosity and Newtonian behavior. The microfluidization process may result in the deterioration of sodium alginate, which could lead to a reduction in the molecular weight of the alginate chains and the viscosity of PPEO nanoemulsions. The degradation may be caused by shear stress, cavitation, and turbulence, which can lead to chemical and physical changes in the material [68,70]. Furthermore, the decrease in droplet size promotes lower interaction between them, also contributing to Newtonian behavior.

The apparent viscosities of the samples after microfluidization were significantly lower than those observed for coarse emulsions (Table 3), which may be attributed to the reduced interaction between the droplets. The apparent viscosity values were obtained at a shear rate of 10.8 s^−1^, as this allows observation of the influence of forces of attraction and repulsion between dispersed droplets on viscosity, thus facilitating understanding of the rheological behavior of the samples in states of slow flow or rest, which are quite common in the food industry.

The rheological properties of sodium alginate dispersions depend on their possibility of forming intermolecular bonds, such as hydrogen bonds, with the viscosity directly linked to polymer-solvent interactions, in addition to factors such as alginate size, structure, and flexibility [68,71]. For instance, larger alginate molecules or those with more complex structures may exhibit higher viscosities due to increased resistance to flow. Similarly, the flexibility of polymer chains can affect their ability to align and flow in response to shear forces, thereby influencing the viscosity [72]. Considering that the viscosity of coarse emulsions is much higher than that of nanoemulsions that have the same composition, the difference in rheological behavior may be associated with the greater interaction between alginate molecules under conditions of exposure to lower mechanical forces (unbroken alginate clusters) or greater ease of interaction between polysaccharide molecules on larger droplets (greater proximity between droplets).

The low viscosity and Newtonian behavior of nanoemulsions represent characteristics that render these systems interesting for the food and pharmaceutical industries, allowing the addition of nanoemulsions to improve the texture and mouthfeel without affecting the rheological properties of foods. In the pharmaceutical field, these characteristics make nanoemulsions optimal delivery systems for controlled-release drugs, in addition to improving the absorption and spreadability of compounds added to cosmetics [73]. An understanding of the rheological properties of emulsions and nanoemulsions allows for the enhancement of their performance and functionality. Furthermore, it facilitates the development of novel, stable, and high-quality foods that align with the demands of the food market and consumers.

### 3.4. Emulsion Stability

**Figure 6:**
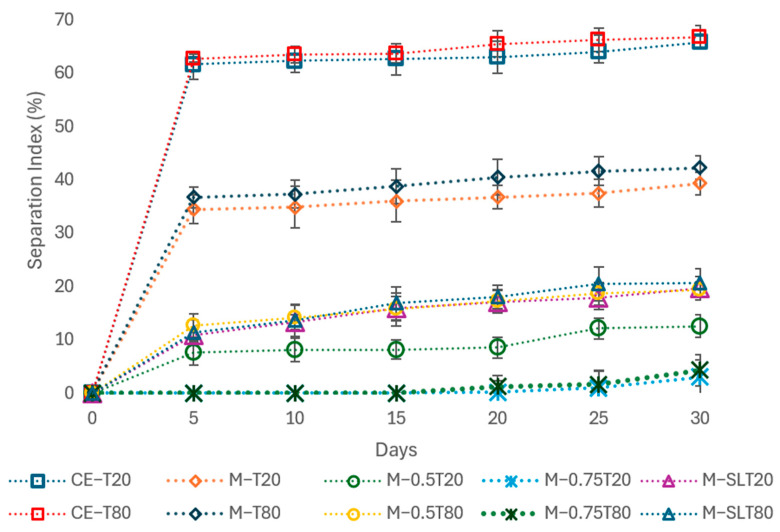
Separation index (SI) of coarse emulsions (CE) and nanoemulsions (M). CE: coarse emulsion; M: microfluidized; T80: Tween 80; T20: Tween 20; 0.5: 0.5% (*w*/*w*) sunflower oil; 0.75: 0.75% (*w*/*w*) sunflower oil; SL: soy lecithin. The data are expressed as means values ± standard deviation.

The stability of emulsions and nanoemulsions is an important parameter for the effective encapsulation of bioactive compounds such as essential oils. The formation of an oil layer, gravitational separation, indicates instability. The curves showing the phase separation indices of the samples are shown in Figure 6.

**Figure 7:**
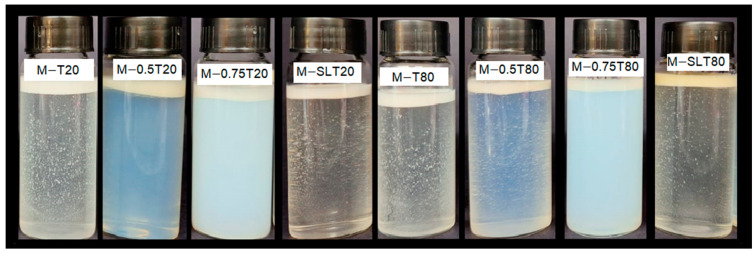
Phase separation of sodium alginate-pink pepper essential oil (PPEO) microfluidized emulsions and nanoemulsions after 30 days of storage. M: microfluidized; T80: Tween 80; T20: Tween 20; 0.5: 0.5% (*w*/*w*) sunflower oil; 0.75: 0.75% (*w*/*w*) sunflower oil; SL: soy lecithin.

At the end of 30 days of storage, despite their formulations, the samples showed phase separation ranging from 3.02 to 62.65% (Figure 7). During the first 20 days of storage, only samples M-0.75T20 and M-0.75T80 had no phase separation. The greater stability of these samples may be related to the addition of sunflower oil combined with their reduced droplet size and PdI values.

The coarse emulsions, CE-T20 and CE-T80, and the microfluidized emulsions, M-T20 and M-T80, showed higher phase separation rates, ranging from 34 to 62%. The high SI values indicate the low stability of the samples, which may be correlated to their larger droplet sizes and PdI. These samples showed instability right after microfluidization, which is the result of the droplets migrating to the top of the emulsion [18]. According to McClements [53], phase separation in emulsions is the result of an increase in droplet size caused by the phenomena of flocculation and coalescence. Guerra-Rosas et al. [74] observed different separation rates according to the essential oil used in the nanoemulsion. While the nanoemulsions with lemongrass and mandarin essential oil showed no phase separation over 56 days of storage, the samples with thyme and oregano essential oil showed separation rates of 6.5 and 17.08%, respectively, indicating that the composition plays a fundamental role in the stability of emulsions and nanoemulsions.

**Figure 8:**
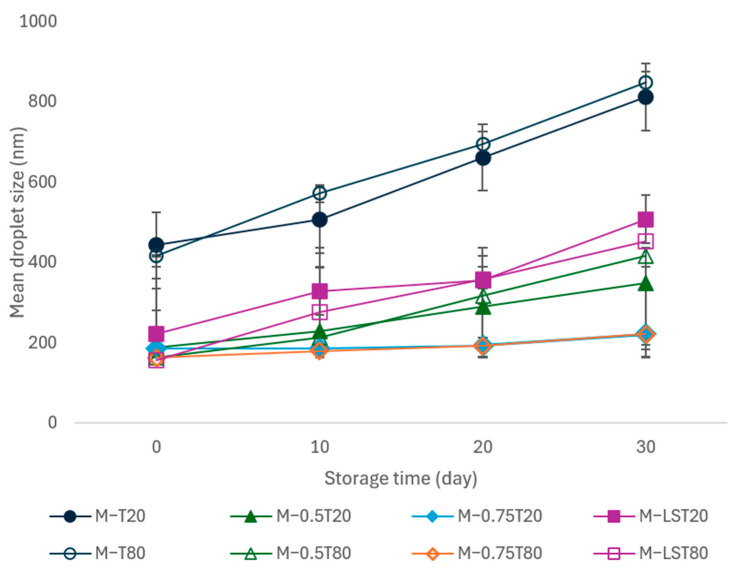
Storage stability of sodium alginate-pink pepper essential oil (PPEO) microfluidized emulsions and nanoemulsions at 25 °C. M: microfluidized; T80: Tween 80; T20: Tween 20; 0.5: 0.5% (*w*/*w*) sunflower oil; 0.75: 0.75% (*w*/*w*) sunflower oil; SL: soy lecithin. The data are expressed as means values ± standard deviation.

The physical stability of nanoemulsions is essential for their commercial use, ensuring that there are no changes in droplet size or phase separation [75]. Figure 8 presents the evolution over time of the mean droplet diameter in emulsions and nanoemulsions stored at 25 °C for 30 days. After microfluidization, all samples except M-0.75T20 and M-0.75T80 showed a significant increase in droplet size. In particular, samples M-T20 and M-T80 showed a significant increase in droplet size and phase separation in the first 24 h, with a stability index (SI) of about 40% after 30 days. Samples containing soy lecithin and 0.5% sunflower oil showed similar behavior, but the SI was reduced to about 20% at the end of the storage period. The destabilization of nanoemulsions is attributed to the Ostwald ripening phenomenon, where the droplet size increases due to the diffusion of smaller oil droplets into larger ones through the aqueous phase. The addition of Ostwald ripening inhibitors, such as sunflower oil and soybean lecithin, can be an effective strategy to improve the stability of nanoemulsions [76]. In this study, the addition of 0.75% sunflower oil conferred greater stability to samples M-0.75T20 and M-0.75T80, which showed only a slight increase in droplet size over 30 days and remained without phase separation for up to 20 days. A similar result was described by Wan et al. [77], who observed no significant changes in the droplet size of clove oil nanoemulsions with corn oil (75% *w*/*w*) over 30 days of storage at 4 and 25 °C. The stability of nanoemulsions is closely related to their small droplet size and PdI values, indicating that these nanoemulsions are resistant to droplet growth caused by coalescence, flocculation, and Ostwald ripening [78]. Ensuring this stability is critical to maintaining the integrity of the active ingredients and ensuring the efficacy of the nanoemulsions, resulting in superior-quality products.

### 3.5. Microscopy

**Figure 9:**
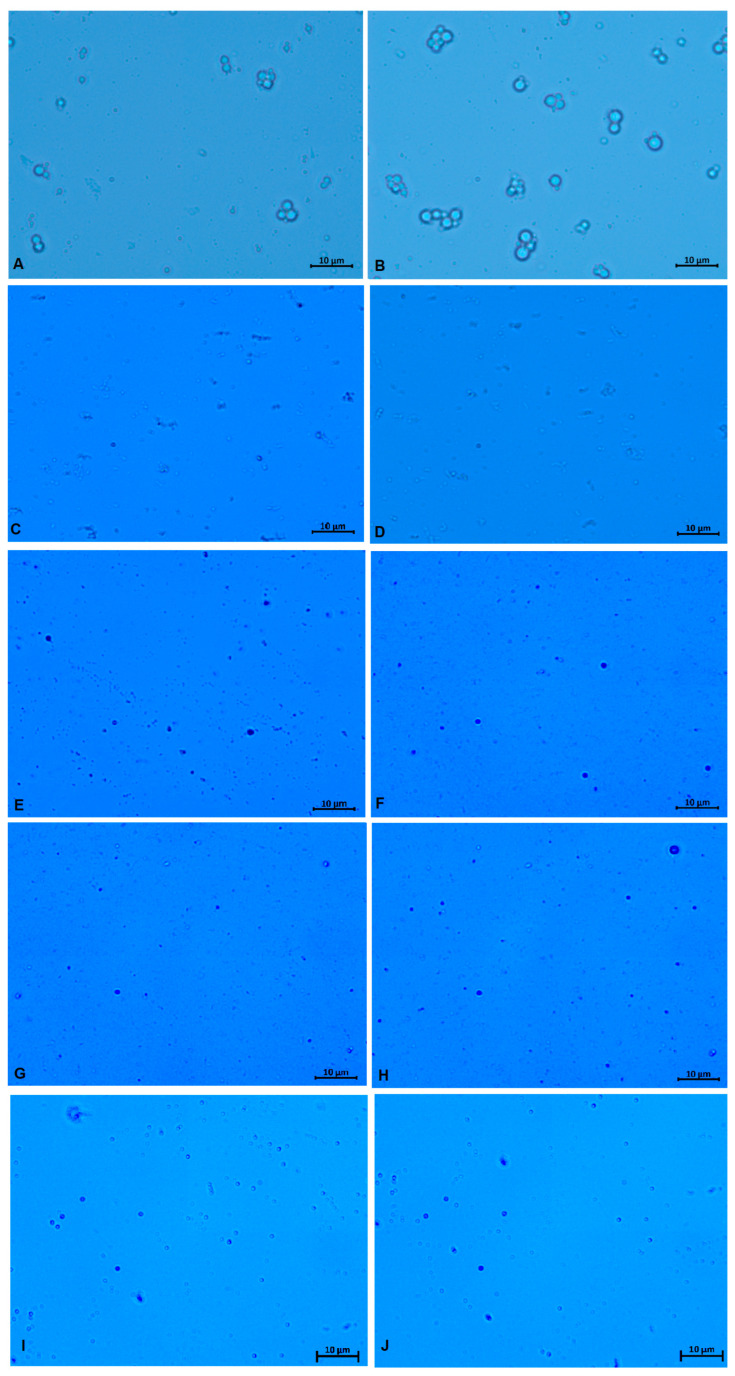
Optical microscopic images of coarse emulsion and sodium alginate-PPEO nanoemulsions. (**A**): CE-T20; (**B**): CE-T80; (**C**): M-T20; (**D**): M-T80; (**E**): M-0.5T20; (**F**): M-0.5T80; (**G**): M-0.75T20; (**H**): M-0.75T80; (**I**): M-SLT20, and (**J**): M-SLT80. CE: coarse emulsion; M: microfluidized; T80: Tween 80; T20: Tween 20; 0.5: 0.5% sunflower oil; 0.75: 0.75% sunflower oil; SL: soy lecithin.

The optical microscopy images of the emulsions and nanoemulsions are shown in Figure 9. The coarse emulsions are shown in images A and B, and it is possible to see the larger droplet size and the aggregation of these droplets. Larger droplets and aggregation lead to the instability of the emulsions, which can induce creaming and phase separation. Sodium alginate-PPEO nanoemulsions can be observed in images B to J, and it is possible to observe a reduction in nanoemulsion droplet size after microfluidization. The samples M-T20 and M-T80 exhibited a larger droplet size distribution and lower pink pepper essential oil retention, and their micrographs (C and D) evidence a higher occurrence of aggregates. The limited accuracy of the images obtained is due to the tiny droplets since accurate observation using optical microscopy techniques diminishes notably when their size falls below 500 nanometers [79].

### 3.6. Oil Retention

**Figure 10:**
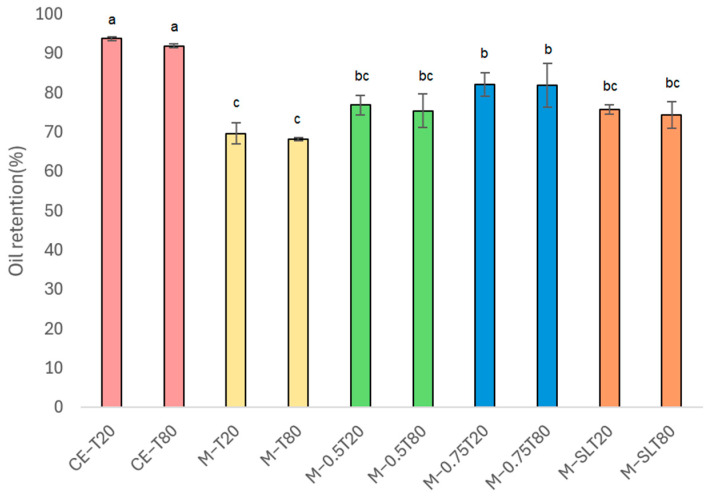
Oil retention of coarse emulsions (CE) and nanoemulsions (M). Significant differences within each column are indicated by different letters (*p* < 0.05) by the Tukey test. CE: coarse emulsion; M: microfluidized; T80: Tween 80; T20: Tween 20; 0.5: 0.5% (*w*/*w*) sunflower oil; 0.75: 0.75% (*w*/*w*) sunflower oil; SL: soy lecithin. The data are expressed as means values ± standard deviation.

Figure 10 shows that the oil content decreased after the microfluidization process. Pink pepper oil-loaded nanoemulsions exhibited oil retention ranging between 68.48% and 82.16%, differing significantly (*p* < 0.05) from the coarse emulsion, which showed oil retention (OR) values greater than 90%. Nanoemulsions M-T80 and M-T20 exhibited the lowest OR values (around 70%). Oil retention is closely associated with droplet size, as higher OR values are associated with smaller droplet sizes [20]. Furthermore, when an essential oil has high water solubility, it displays a tendency to partition predominantly into the aqueous phase. This phenomenon results in a reduction in encapsulation efficiency because a portion of the essential oil may be lost to the aqueous phase and may not be effectively nano-encapsulated within the oil droplets of the nanoemulsion [24]. However, considering the above, coarse emulsions should have lower OR compared to M-T80 and M-T20 nanoemulsions. Although microfluidization produces stable and uniform nanoemulsions, it can also impact the oil retention within these systems. The high shear rates and elevated temperatures during the process may compromise droplet structure and increase diffusion rates, leading to the loss of encapsulated oil [80]. By understanding these challenges and optimizing process conditions, such as controlling shear rates and temperature, along with proper formulation, essential oil retention can be improved, thereby enhancing the effectiveness of nanoemulsions in various applications.

The addition of soy lecithin to the composition of the emulsions (M-SLT20 and M-SLT80) increased the OR to values above 75%. This protective effect is due to the ability of lecithin to form hydrogen bonds between its phosphate groups and the hydroxyl groups of the essential oils [55]. Lecithin aliphatic phospholipid chains envelop the essential oil, effectively trapping it within the nanoemulsion structure [58,81]. Nanoemulsions containing 0.75% (*w*/*w*) of sunflower oil showed even higher encapsulation levels, ranging from 81.91% to 82.16%. Dávila-Rodrigues et al. [20] observed 82% retention of oregano essential oil after producing a nanoemulsion by high-frequency ultrasound. However, the amount of *Coriandrum sativum* essential oil in the Das et al. [82] nanoemulsion produced by ultrasonication ranged from 26 to 76%. The difference in the oil content of the nanoemulsions can be directly related to losses in the process since essential oils are volatile, and high-energy processes generate heat that can induce the volatilization of these oils. According to Niu et al. [83], nanoemulsions with encapsulation efficiency above 80% are considered highly stable. The incorporation of essential oils into vegetable oil, such as sunflower oil, offers protection against microfluidization heating, minimizing its degradation and volatilization [84]. The lipophilic nature of sunflower oil acts as a protective matrix, increasing solubility and reducing exposure to aqueous phases where temperature fluctuations occur [85]. The protection mechanisms provided by both sunflower oil and lecithin contribute to the stability and integrity of the encapsulated essential oils in the nanoemulsion system.

The hydro distillation process, commonly used to extract essential oils from plant materials, can also be applied to extract oil from emulsified systems. This process involves three main physicochemical processes: hydrodiffusion, hydrolysis, and thermal decomposition, which can significantly affect the retention of oil in the system [86]. The oil retention rate is closely linked to the stability of the emulsions and the conditions of the process. As heating is applied, the stability of the emulsions may decrease, leading to coalescence and phase separation, which releases oil from the structure, ultimately reducing oil retention. Additionally, volatilization and thermal degradation of oil components can occur, leading to evaporation losses and alterations in the chemical composition of the oil [22,87,88]. Therefore, the greater the stability of the emulsion, the higher the amount of oil retained in the system. The retention of oil in the nanoemulsion is directly influenced by the interaction between the components of the formulation, such as surfactant, oil, polymer, and fabrication method, which will directly affect the stability and release properties of the nanoemulsion [22]. Therefore, the right choice of manufacturing process and formulation can guarantee the development of a nanoemulsion with the right properties for its purpose.

### 3.7. Antimicrobial Activity

**Figure 11:**
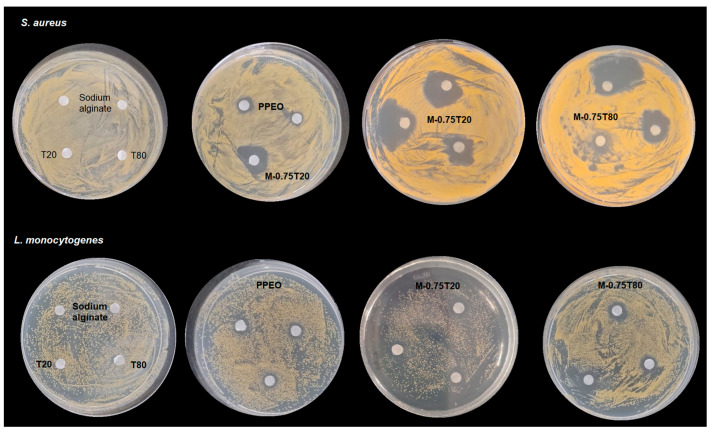
Antimicrobial activity of non-encapsulated PPEO and its encapsulated forms, as well as Tween 20, Tween 80, and sodium alginate solution against *S. aureus* and *L. monocytogenes*. PPEO: pink pepper essential oil (non-encapsulated); M: microfluidized; T80: Tween 80; T20: Tween 20; 0.75: 0.75% (*w*/*w*) sunflower oil.

The antimicrobial activity of pure PPEO (non-encapsulated) and its nanoemulsions was investigated against gram-positive bacteria, *Staphylococcus aureus*, *Listeria monocytogenes*, and gram-negative bacteria, and *Salmonella* sp. and *Escherichia coli*. Disk diffusion test results are shown in Table 4. Sodium alginate, Tween 20, and Tween 80 showed no antimicrobial activity against the microorganisms evaluated. PPEO and two nanoemulsions, M-0.75T80 and M-0.75T20, showed antimicrobial action against the gram-positive bacteria *L. monocytogenes* and *S. aureus*, while *E. coli* and *Salmonella* sp. were not sensitive (Figure 11). Pure PPEO was previously tested by Dannenberg et al. [9] against *L. monocytogenes*, *S. aureus*, *E. coli*, and *S*. Typhimurium and exhibited antimicrobial activity exclusively against the gram-positive bacteria *L. monocytogenes* and *S. aureus* with inhibition zones of 24.30 and 23.09 mm, respectively (similar to our results). The heightened susceptibility of gram-positive bacteria to PPEO is attributed to the facile penetration of nanometer-scale hydrophobic molecules through the peptidoglycan layer that constitutes the structure of gram-positive bacteria. However, the double layer of lipopolysaccharide found in gram-negative bacteria acts as a defense mechanism to the passage of antimicrobial molecules [89,90]. However, Salvia-Trujillo et al. [33] documented the inhibition of *E. coli* with sodium alginate nanoemulsion loaded with lemongrass and clove oil produced in a microfluidization process. The results demonstrated that the antimicrobial effect is predominantly influenced by the active compounds of essential oils rather than their concentration [91].

MIC and MBC values of samples that exhibited antimicrobial activity in the disk diffusion test are presented in Table 5. The differences in the inhibition halos and MIC and MBC values were greater than those exhibited by PPEO (non-encapsulated). The antimicrobial activity of nanoemulsions is associated with several factors, including oil retention (approximately 82% in emulsions with 0.75% sunflower oil), the small average droplet size of nanoemulsions (160.7–186.6 nm), and their stability. The reduction in droplet size of nanoemulsions enhances the functionality of EOs due to the increase in the surface area of the droplets [74]. Some authors have reported that encapsulation of essential oil (EO) can increase their antimicrobial activity [34,92,93]; however, in other cases, this activity may be reduced [75,94], indicating that the antimicrobial efficacy of nanoemulsions-based EO can be significantly affected due to the influence of EO components, formulation, and the nanoemulsion droplet size [90].

## 4. Conclusions

Different sodium alginate-PPEO nanoemulsions were produced via microfluidization, and the findings demonstrated a notable influence of this process on their physicochemical, rheological, and antimicrobial properties. Sodium alginate-PPEO nanoemulsions showed small droplet size, multimodal particle size distribution, and high (absolute) and negative zeta potential values. Among the eight formulations outlined in the current study, M-0.75T80 and M-0.75T20 showed remarkable performance concerning the evaluated properties. The incorporation of sunflower oil into the formulation improved the stability of nanoemulsions during long storage and led to an increase in the PPEO retention within the nanoemulsion. The sodium alginate-PPEO nanoemulsion demonstrated remarkable antimicrobial activity against *L. monocytogenes* and *S. aureus*, which was associated with the formulation, oil retention, and droplet size.

Nevertheless, the production of nanoemulsions via microfluidization represents a significant challenge, primarily due to the elevated costs associated with the requisite equipment and the necessity for precise processing conditions to guarantee the stability and uniformity of emulsions on a large scale. These challenges need to be addressed to facilitate the commercial application of these nanoemulsions. Even so, the results obtained are noteworthy due to the promising advantages of using nanoemulsions as delivery systems, with potential for applications in food packaging. Future investigation is required to ascertain the efficacy of these nanoemulsions as coatings in enhancing food quality and prolonging the shelf life of foods. This will be crucial to elucidate their real potential for incorporation into food products.

## Figures and Tables

**Table 1:** Composition and identification of the different nanoemulsion formulations.

Sample	PPEO (%*w*/*w*)	Sodium Alginate (%*w*/*w*)	T20 (%*w*/*w*)	T80 (%*w*/*w*)	Sunflower Oil (%*w*/*w*)	SL (%*w*/*w*)
T20	1.0	1.0	1.0	-	-	-
0.5T20	1.0	1.0	1.0	-	0.5	-
0.75T20	1.0	1.0	1.0	-	0.75	-
SLT20	1.0	1.0	1.0	-	-	0.2
T80	1.0	1.0	-	1.0	-	-
0.5T80	1.0	1.0	-	1.0	0.5	-
0.75T80	1.0	1.0	-	1.0	0.75	-
SLT80	1.0	1.0	-	1.0	-	0.2

PPEO: pink pepper essential oil; T20: Tween 20; T80: Tween 80; and SL: soy lecithin.

**Table 2:** Mean particle size (z-average, D10, D50, and D90) and polydispersity index (PdI) of coarse emulsions (CE) and nanoemulsions (M).

Samples	Z-Average (nm)	Particle Size (nm)			PdI
		D_90_	D_50_	D_10_	
CE-T20	1323 ± 156 ^a^	1950 ± 1 ^a^	804 ± 6 ^b^	59.3 ± 5.5 ^b^	0.891 ± 0.168 ^a^
CE-T80	998 ± 29 ^b^	1570 ± 101 ^b^	1010 ± 10 ^a^	87.7 ± 3.9 ^a^	0.830 ± 0.140 ^a^
M-T20	443 ± 10 ^c^	468 ± 23 ^c^	214 ± 5 ^c^	103 ± 2 ^c^	0.568 ± 0.091 ^b^
M-T80	434 ± 27 ^c^	498 ± 16 ^c^	229 ± 8 ^c^	113 ± 4 ^c^	0.638 ± 0.073 ^b^
M-0.5T20	221 ± 2 ^d^	229 ± 15 ^de^	106 ± 8 ^e^	61.4 ± 3.6 ^e^	0.303 ± 0.005 ^c^
M-0.5T80	162 ± 2 ^d^	298 ± 6 ^d^	126 ± 7 ^d^	65.4 ± 5.1 ^d^	0.367 ± 0.044 ^c^
M-0.75T20	187 ± 3 ^d^	198 ± 12 ^de^	30.1 ± 4.0 ^f^	18.3 ± 2.6 ^f^	0.294 ± 0.010 ^c^
M-0.75T80	161 ± 4 ^d^	175 ± 10 ^de^	26.6 ± 3.5 ^f^	18.4 ± 4.5 ^f^	0.273 ± 0.007 ^c^
M-SLT20	183 ± 3 ^d^	257 ± 16 ^de^	34.3 ± 4.1 ^f^	22.7 ± 4.7 ^f^	0.411 ± 0.060 ^c^
M-SLT80	160 ± 5 ^d^	213 ± 9 ^de^	29.8 ± 2.6 ^f^	21.0 ± 4.3 ^f^	0.391 ± 0.039 ^c^

Significant differences within each column are indicated by different letters (*p* < 0.05) by the Tukey test. CE: coarse emulsion; M: microfluidized; T80: Tween 80; T20: Tween 20; 0.5: 0.5% (*w*/*w*) sunflower oil; 0.75: 0.75% (*w*/*w*) sunflower oil; SL: soy lecithin. The data are expressed as means values ± standard deviation.

**Table 3:** Viscosity (m Pa·s) and rheological properties of coarse emulsions (CE) and nanoemulsions (M) at 10.8 s^−1^ from the power-law model.

Samples	Viscosity (mPa·s)	*n*	*k* (Pa·s^n^)	R^2^
CE-T20	172.11 ± 3.18 ^a^	0.727 ± 0.006 ^b^	0.4239 ± 0.0101 ^b^	0.998
CE-T80	172.08 ± 5.68 ^a^	0.727 ± 0.005 ^b^	0.4218 ± 0.0209 ^b^	0.998
M-T20	35.33 ± 0.53 ^b^	0.986 ± 0.002 ^a^	0.0304 ± 0.0011 ^a^	1.000
M-T80	33.45 ± 0.41 ^b^	0.988 ± 0.001 ^a^	0.0286 ± 0.0015 ^a^	1.000
M-0.5T20	31.23 ± 0.43 ^b^	0.990 ± 0.001 ^a^	0.0261 ± 0.0001 ^a^	0.999
M-0.5T80	32.94 ± 0.14 ^b^	0.988 ± 0.001 ^a^	0.0285 ± 0.0001 ^a^	1.000
M-0.75T20	32.36 ± 0.35 ^b^	0.988 ± 0.001 ^a^	0.0274 ± 0.0004 ^a^	1.000
M-0.75T80	33.92 ± 0.51 ^b^	0.988 ± 0.005 ^a^	0.0293 ± 0.0008 ^a^	1.000
M-SLT20	34.54 ± 0.20 ^b^	0.987 ± 0.001 ^a^	0.0293 ± 0.0008 ^a^	0.999
M-SLT80	33.84 ± 0.76 ^b^	0.986 ± 0.002 ^a^	0.0290 ± 0.0009 ^a^	0.999

*k*: consistency coefficient, *n*: flow behavior index. Significant differences within each column are indicated by different letters (*p* < 0.05) by the Tukey test. CE: coarse emulsion; M: microfluidized; T80: Tween 80; T20: Tween 20; 0.5: 0.5% (*w*/*w*) sunflower oil; 0.75: 0.75% (*w*/*w*) sunflower oil; SL: soy lecithin. The data are expressed as means values ± standard deviation.

**Table 4:** Diameters of inhibition zone (mm) demonstrated by non-encapsulated pink pepper essential oil and sodium alginate-PPEO nanoemulsions against both Gram-positive and Gram-negative bacterial strains.

	Inhibition Zone (mm)			
	*S. aureus*	*L. monocytogenes*	*E. coli*	*Salmonella* sp.
PPEO	14.13 ± 0.19 ^a^	12.36 ± 1.90 ^a^	ND	ND
M-T20	ND	ND	ND	ND
M-T80	ND	ND	ND	ND
M-0.5T20	ND	ND	ND	ND
M-0.5T80	ND	ND	ND	ND
M-0.75T20	26.18 ± 3.87 ^b^	17.13 ± 0.73 ^ab^	ND	ND
M-0.75T80	26.57 ± 6.99 ^b^	23.28 ± 4.50 ^b^	ND	ND
M-SLT20	ND	ND	ND	ND
M-SLT80	ND	ND	ND	ND

Significant differences within each column are indicated by different letters (*p* < 0.05); PPEO: pink pepper essential oil (non-encapsulated); M: microfluidized; T80: Tween 80; T20: Tween 20; 0.5: 0.5% (*w*/*w*) sunflower oil; 0.75: 0.75% (*w*/*w*) sunflower oil; SL: soy lecithin. The data are expressed as means values ± standard deviation; ND = Not Detected.

**Table 5:** Values for the minimal inhibitory concentration (MIC) and minimal bactericidal concentration (MBC) values (μg/mL) pink pepper essential oil and sodium alginate-PPEO nanoemulsions.

	*L. monocytogenes*		*S. aureus*	
	MIC (µg/mL)	MBC (µg/mL)	MIC (µg/mL)	MBC (µg/mL)
PPEO	200	800	400	1600
M-0.75T20	200	400	200	800
M-0.75T80	200	400	200	800

PPEO: pink pepper essential oil (non-encapsulated); M: microfluidized; Tween 80; T20: Tween 20; 0.75: 0.75% (*w*/*w*) sunflower oil.

## Data Availability

The data provided in this study are available on request from the corresponding author. The data are not publicly accessible due to privacy considerations.

## Supplementary Materials

The following resources are available for online consultation at https://www.mdpi.com/article/10.3390/foods13193090/s1, Figure S1: Intensity and volume-particle size distribution (nm) of the alginate-PPEO coarse emulsion (CE) stabilized by Tween 20 and Tween 80.
