# Supplementary material for: Physicochemical Characterization, Rheological Properties, and Antimicrobial Activity of Sodium Alginate-Pink Pepper Essential Oil (PPEO) Nanoemulsions
Source: Foods. 2024 Sep 27;13(19):3090. doi: 10.3390/foods13193090 (PMC11476015; doi:10.3390/foods13193090)
Supplement: Supplementary file 1 [file foods-13-03090-s001.zip › foods-3163732-supplementary.pdf]

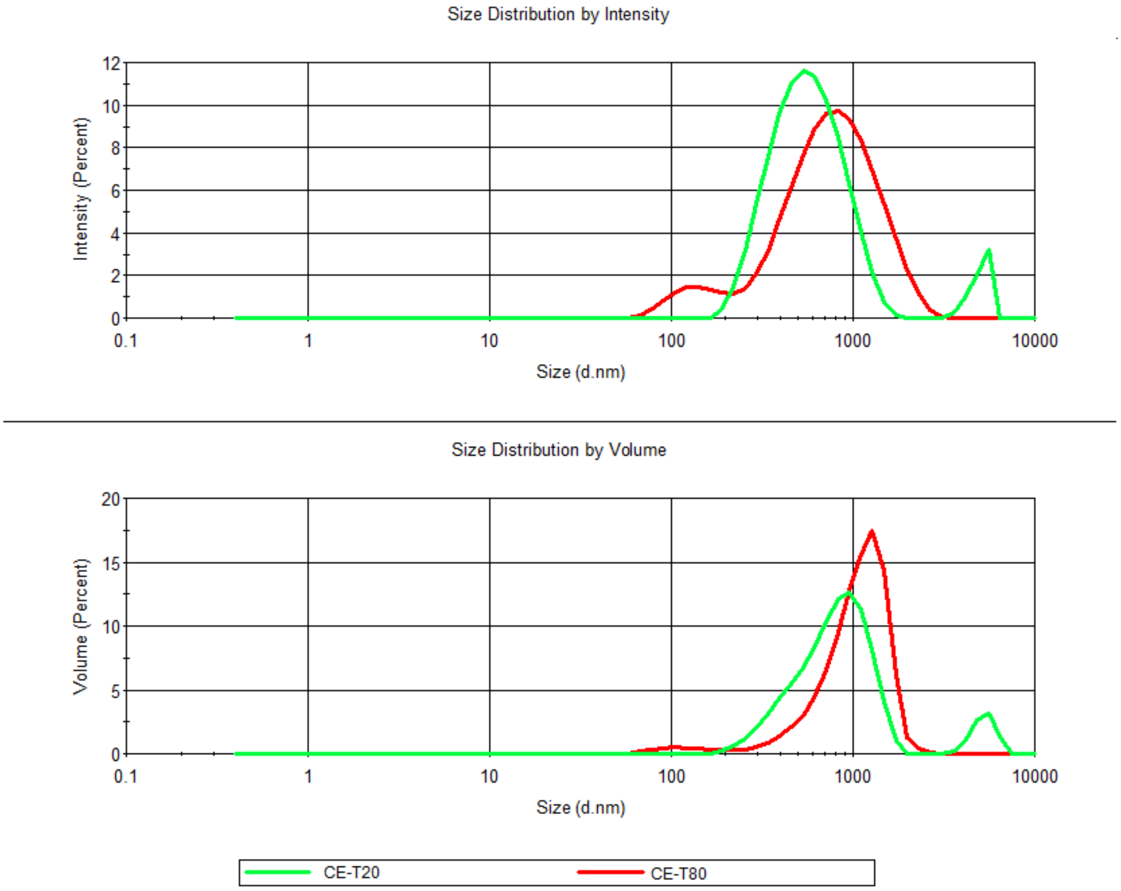

Figure S1 - Intensity- and volume-particle size distribution (nm) of the alginate-PPEO coarse emulsion (CE) stabilized by Tween 20 and Tween 80. T80: Tween 80; T20: Tween 20.
